# Arthroscopic Treatment of Popliteus Tendinitis Using the Accessory Portal

**DOI:** 10.3389/fsurg.2022.860300

**Published:** 2022-04-21

**Authors:** B. Chen, H. K. Liu, H. Wang

**Affiliations:** ^1^ Division of Joint Surgery and Sports Medicine, Department of Orthopedic Surgery, Zhongnan Hospital of Wuhan University, Wuhan, Hubei Province, China; ^2^ Department of Pain, The Eighth Affiliated Hospital of Sun Yat sen University, Shenzhen, Guangdong Province, China

**Keywords:** arthroscopic treatment, popliteus tendinitis, extreme lateral approach, popliteus tendon, diagnosis

## Abstract

**Introduction:**

This study aimed to evaluate the effect of arthroscopic treatment of popliteus tendinitis via an auxiliary extreme lateral approach and to investigate the pathogenesis and treatment of popliteus tendinitis.

**Materials and Methods:**

From 2016 to 2020, arthroscopic popliteus tendon ablation was performed in 15 patients (15 knees) with popliteus tendinitis via an auxiliary extreme lateral approach. Clinical outcomes were assessed using the Lysholm knee scoring scale, the Tegner score, the International Knee Documentation Committee (IKDC) score and the visual analogue scale (VAS) pain score at the 24-month follow-up after surgery.

**Results:**

A total of 15 patients (mean age, 51.1 ± 7.1 years) were included. They had a mean body mass index of 23.8 ± 2.1 kg/m^2^. The minimum follow-up period was 24 months. Comparing the postoperative state to the preoperative state, the mean postoperative Lysholm score, Tegner score, and IKDC score improved significantly from 70.0 ± 5.0, 3.0 ± 0.9, and 62.3 ± 5.5 to 89.3 ± 4.2, 4.6 ± 0.61, and 80.5 ± 4.4, respectively (*p* < 0.01). The preoperative VAS score for pain improved from 6.4 ± 0.5 to 0.9 ± 0.4 (*p* < 0.01). No patients were lost to follow-up.

**Conclusions:**

Following arthroscopic-assisted treatment, all the patients with popliteus tendinitis achieved satisfactory clinical outcomes in terms of pain relief and improved function.

**Level of Evidence:**

Level IV

## Introduction

With medical diagnostic and technical improvements, recent studies indicated that popliteus tendon injuries were reported with increasing frequency due to the popularity of higher levels of sports (1). However, popliteus tendinitis was an uncommon pathology that often occurs in professional athletes and dancers (2–4). Patients with this condition usually had posterolateral pain in the knee joint. The symptoms of popliteus tendinitis were severe enough to limit or prevent athletic participation, thereby affecting daily life and work. Because the pain was very close to the joint line, sometimes patients were misdiagnosed as having a meniscal tear or lateral compartment articular damage and occasionally treated with arthroscopic meniscus partial resection or arthrotomy (3). On the other hand, many physicians lacked professional knowledge about popliteus tendinitis, leading to inaccurate diagnoses. Therefore, mastering knowledge about the disease and learning how to manage it correctly are essential.

Many non-surgical options were described for the treatment of symptomatic popliteus tendinitis, including strengthening of the quadriceps muscle, rest, NSAIDs (Nonsteroidal Antiinflammatory Drugs), and localized corticosteroid injections (5). With these conservative treatments, the majority of patient symptoms was significantly relieved, and the patient recovered the ability to perform normal physical exercise. However, there were certain drawbacks to these treatments (6). That is, the patient symptoms were prone to relapse. Drugs had side effects, such as allergies and impairment of liver and kidney function. Furthermore, repeated corticosteroid injections resulted in reduced biomechanical performance of the popliteus tendon (7). In view of this situation, there is an urgent need to find an updated solution to this dilemma.

In this article, we described a novel arthroscopic technique that treated recurrent and refractory popliteus tendinitis. Clinical data for patients with popliteus tendinitis were retrospectively analyzed. The patients followed up for at least 24 months after surgery. We speculated that popliteus tendon could be better observed through this kind of procedure, which also helped in the diagnosis of popliteus tendinitis. Besides, under this operation, these patients achieved better clinical effects.

## Materials and Methods

### Patients

From 2016 to 2020, we retrospectively collected fifteen patients (15 knees) in our study. The 15 patients were clearly diagnosed with popliteus tendinitis and treated by arthroscopic interventions via an auxiliary extreme lateral approach. There were no cases of calcific tendinitis of the popliteus tendon. Approval for the study was granted by the Ethics Committee of Zhongnan Hospital of Wuhan University.

**Figure 1:**
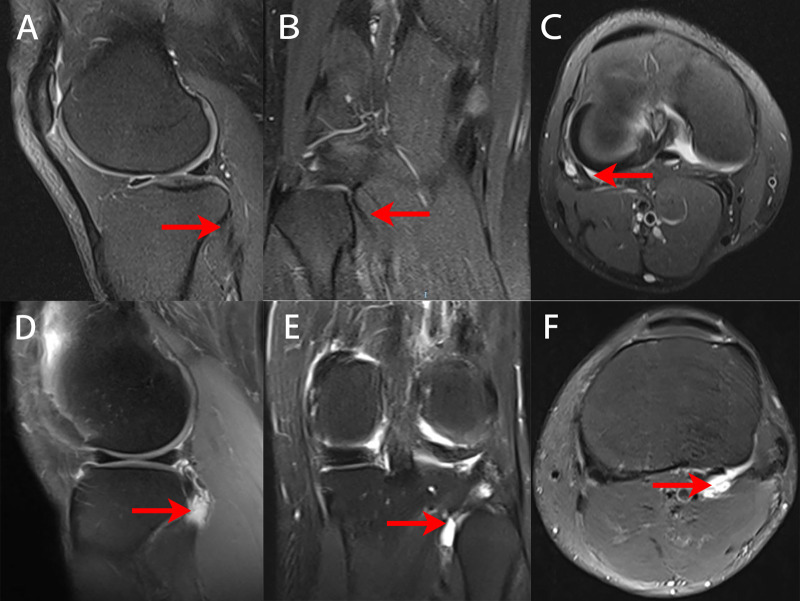
MRI images of the knees of normal people and of patients with popliteus tendinitis. Red arrows show the location of the popliteus tendon. (**A**–**C**) Preoperative sagittal, coronal and horizontal MRI views of MRI of the normal people. (**D**–**F**) Preoperative sagittal, coronal and horizontal MRI views of a 55-year-old female with popliteus tendinitis.

The inclusion criteria were as follows: (1) All patients had a chronically painful (at least one-year history) of posterolateral compartment that was unresponsive to at least two conservative treatment sessions (physical therapy, rest and extracorporeal shock wave therapy) plus at least two local anesthetic-steroid injections. (2) No patients had any history of knee surgery and infection. (3) All patients followed up for at least 24 months after surgery. (4) The patients had indispensable imaging findings, such as MRI and radiographs, before the operation (**Figure 1**). (5) All operations were performed via an auxiliary extreme lateral approach by the same experienced surgeon. The exclusion criteria were as follows: (1) The patients had the lateral meniscal tear or lateral compartment articular damage. (2) The patients had loose bodies of knee. (3) The patients had malignancy or other concomitant rheumatic inflammatory conditions.

### Physical Examination

**Figure 2:**
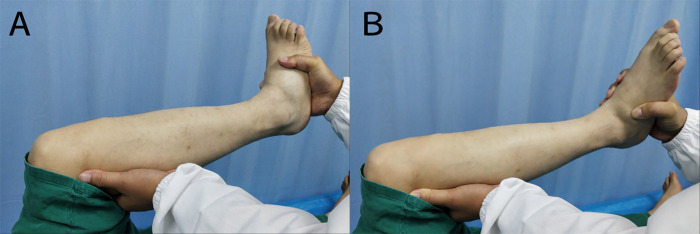
(**A**) Garrick test (active tibial external rotation against resistance). (**B**) Passive external rotation test.

Each patient received regular physical examinations of the knee joint and a specific exam for the popliteus tendon. In addition, there were two tests aimed at isolating and diagnosing issues with the popliteus muscles and evaluating popliteus tendon injury (**Figure 2**): (1) Garrick test (active tibial external rotation against resistance): the patient was in the supine position, the hip and knee were flexed to 90°, and the leg as internally rotated. A symptomatic patient experiences pain with resisted tibial internal rotation (5). (2) Passive external rotation test: the patient was asked to apply passive external force with the leg in the same position (5).

### Surgical Technique

**Figure 3:**
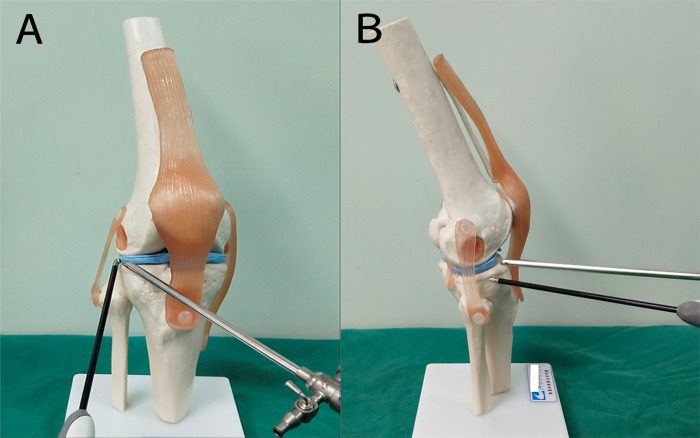
Model of an auxiliary extreme lateral approach combined with a conventional anterolateral approach in the knee. (**A**) anterolateral view, (**B**) lateral views.

First, standard anterolateral (AL) and anteromedial (AM) portals were established to explore the intact intra-articular structure and the posterior horn of the lateral meniscus in particular. Second, hyperplasia of the synovium, synovial plica, edema, wear of the cruciate ligaments or meniscus as well as osteochondral injury were detected and approached simultaneously. Finally, an extreme lateral approach was established to examine and assess the popliteus hiatus and the status of the popliteus tendon (**Figure 3**).

**Figure 4:**
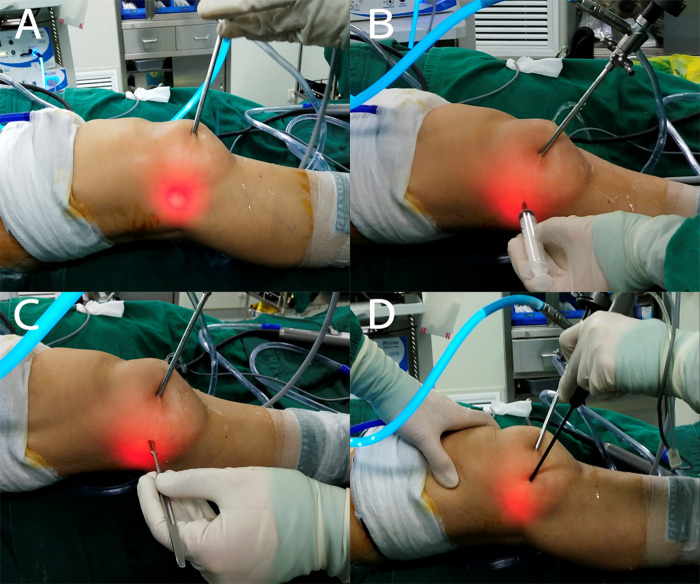
(**A**–**D**) The location and establishment of an auxiliary extreme lateral approach.

**Figure 5:**
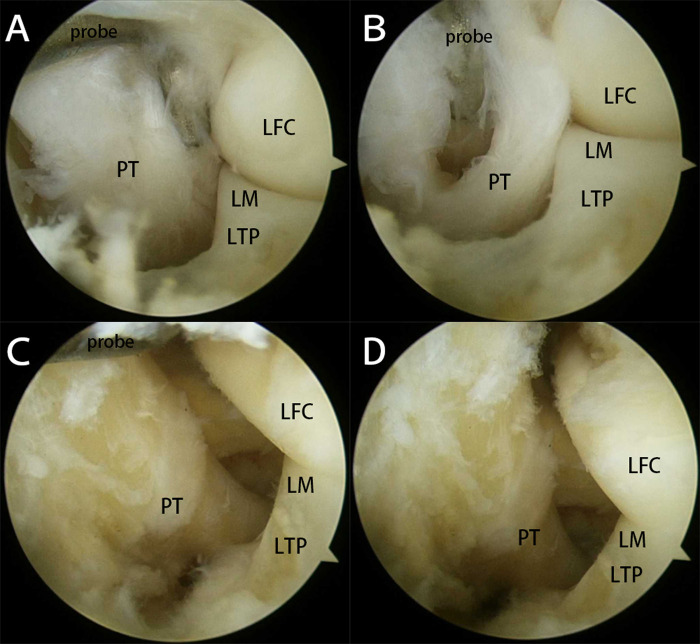
The arthroscopic finding of the popliteus tendon and popliteus hiatus via an auxiliary extreme lateral approach. (**A**,**B**) Before ablation: The arthroscopic finding of the popliteus tendon and popliteus hiatus (PT, popliteus tendon; LM, lateral meniscus; LFC, lateral femoral condyle; LTP, lateral tibial plateau). (**C**,**D**) After ablation: The arthroscopic finding of the popliteus tendon and popliteus hiatus (PT, popliteus tendon; LM, lateral meniscus; LFC, lateral femoral condyle; LTP, lateral tibial plateau).

The extreme lateral approach was approximately 1 cm above the joint line and 3 cm outside the anterolateral portal. The arthroscope was introduced via the AL portal. Using the arthroscope’s light to aid in positioning, an injector needle was percutaneously inserted at a site between the AL portal and the posterolateral (PL) portal. After seeing the needle under arthroscopy, a small stab wound was made using a No. 11 blade at the puncture site and enlarged with a straight hemostat. This approach was established under arthroscopy with the full knee extension (**Figure 4**). Through this approach, the popliteus hiatus and coverage of the popliteus tendon could be clearly viewed during extension and flexion of the knee joint (**Figure 5**). We used plasma ablation to ablate the inflamed part of the popliteal tendon under arthroscopy. At the same time, we also ablated and shaved the inflammatory synovial tissue surrounding the popliteal tendon appropriately (Supplementary Video S1).

### Postoperative Management

Postoperative management included diminishing pain and swelling and establishing a full range of motion to prevent muscle hypotrophy. First, all patients were treated with rest, ice, compression, and elevation of the injured knee postoperatively. They were full weight bearing immediately, but the time and intensity of their activities were limited one week after the operation. The subsequent therapeutic program focused on restoring the knee joint range of motion, incorporating quadriceps strengthening and progressing to dynamic proprioceptive training. Conditioning was maintained with the use of an exercise bike and walking and, eventually, running and other sport-specific exercises. Patients were discharged the day after surgery and visited the outpatient clinic for follow-up.

### Outcome Evaluation

Clinical and functional evaluations were carried out with preoperative and postoperative Lysholm scores, Tegner scores, IKDC scores and VAS scores. The postoperative scores were used from the 24-month follow-up after surgery.

### Statistical Methods

SPSS 20 statistical software was used for the statistical analysis. Descriptive statistics were used to report the data. Baseline patient characteristics were expressed as *n* for categorical variables and as the mean with the standard deviation for continuous variables. A two-sided paired samples *t* test was used to compare age, BMI, and the Lysholm score, Tegner score, IKDC score, VAS pain scale before and after surgery. A p-value less than 0.05 was considered statistically significant.

## Results

Fifteen patients (15 knees) underwent arthroscopic-assisted treatment for popliteus tendinitis. The cohort comprised 9 females and 6 males with a mean age of 51.1 years (38–63 years). The mean body mass index of participants was 23.8 ± 2.1 kg/m^2^, and the minimum follow-up duration was 24 months.

In this study, all patients had a history of excessive exercise. All of them participated in at least one sports activity every week. They all complained of significant tenderness on the popliteus tendon and had positive signs during at least one specific physical examination. No significant abnormalities were observed on their radiographs. According to the MRI findings, 11 patients had increased intratendinous or myotendinous signals on fluid-sensitive sequences, 12 patients had fluid effusion of the popliteus tendon, and 1 patient had partial degenerative tearing of the popliteus tendon (**Table 1**).

**Table 1:** Diagnosis of popliteus tendinitis.

Case history and physical examination	Patients (*n* = 15)
History of trauma	2
Tenderness along the popliteus tendon	15
Garrick test	11
Passive external rotation test	13
MRI features	
Local tendon enlargement	3
Increased intratendinous or myotendinous signal on fluid-sensitive sequences	11
Unusual amount of fluid around tendon	12
Partial tendon tearing	1

The preoperative Lysholm, Tegner, IKDC and VAS scores were 70.0 ± 5.0, 3.0 ± 0.9, 62.3 ± 5.5, and 6.4 ± 0.5, respectively. The last follow-up Lysholm, Tegner, IKDC and VAS scores were 89.3 ± 4.2, 4.6 ± 0.6, 80.5 ± 4.4, and 0.9 ± 0.4, respectively. There were significant differences between the preoperative and postoperative scores (Lysholm *p* < 0.01, Tegner *p* < 0.01, IKDC *p* < 0.01, VAS *p* < 0.01) (**Table 2**), which indicated good outcomes.

**Table 2:** Pre- and Post-operative Knee Function Scores for Patients Included.

Outcome score (Mean ± SD)	Preoperative	Postoperative	*p* value
Lysholm	70.0 ± 5.0	89.3 ± 4.2	<0.01
Tegner	3.0 ± 0.9	4.6 ± 0.6	<0.01
IKDC	62.3 ± 5.5	80.5 ± 4.4	<0.01
VAS	6.4 ± 0.5	0.9 ± 0.4	<0.01

## Discussion

Our literature review found no previous studies reported arthroscopic surgery for recurrent popliteus tendinitis, which led to our interest in studying this topic. Theoretically, popliteus tendinitis means localized inflammation in the tendon of the popliteus (6, 8). As a kind of tendinitis, popliteus tendinitis also had some common symptoms of tendinitis itself. The main complaint of patient was the pain in the posterolateral corner of the knee. In some cases, there was significant tingling accompanied by sharp pain and joint stiffness, which limited the movement of the affected joints (3, 8). In the cases that we included, most of the patients were middle-aged and elderly individuals. The clinical manifestations of the preoperative patients affected their daily living. At present, many researchers proposed that repeated minor trauma, repetitive application of knee rotation in turnout, exercise, overuse and poor blood supply to tendons were the most common causes of popliteus tendinitis (2, 3, 9).

Based on cadaveric study, LaPrade et al. reported that the popliteus tendon rested proximal to the popliteus sulcus on the lateral femoral condyle from full knee extension to 112° of flexion. At knee angles of 112° or more, the popliteus tendon engaged in the popliteus sulcus (10, 11). However, to date, there had no reports in the literature about the intra-articular trajectory of the popliteus tendon. According to our arthroscopic findings, the popliteus tendon could be comprehensively observed arthroscopically via an auxiliary extreme lateral approach. We found that the popliteus tendon stretched properly and externally rotated against the lateral tibial plateau and the lateral femoral condyle when the knee flexed from full extension. Conversely, when the knee moved from approximately 90° of flexion angle to extension, the popliteus tendon began to internally rotated relative to the lateral tibial plateau and the lateral femoral condyle (Supplementary Video S2). Consequently, we speculated that the mechanism of popliteus tendon injury might be related to repeated rolling friction against the lateral tibial plateau and the lateral femoral condyle, and traction injury to its collagen fiber tissue. Previous research reported that the popliteus tendon was in tension when the proximal tibia was externally rotated. It became relaxed when the tibia was internally rotated (12, 13). Due to the clinically relevant anatomy of the popliteus tendon, Marc et al. found that downhill running and other deceleration activities were prone to lead to popliteus tendinitis. This might stem from the popliteus acting to prevent excessive posterior tibial translation relative to the femur (14). Ferrari et al. indicated that inflammation of the popliteus was associated with overuse or fatigue of the quadriceps (4). When the fatigued quadriceps could not adequately resist forward displacement of the femur on the tibia, undue stress occurred on the secondary restraints and overwhelmed the relatively small popliteus muscle. Moreover, the blood supply of the popliteus muscle is from the medial inferior genicular branch of the popliteus artery and the muscular branch of the posterior tibial artery (15). It is different from the posterior cruciate ligament, which has abundant blood vessel and synovial coverage. There was no obvious capillary and synovial coverage on the surface of the popliteal tendon under the arthroscopic examination. Thus, it occurred with some physical injuries and degeneration over time.

Popliteus tendinitis was not a common cause of knee pain. Physicians were not knowledgeable about popliteus tendinitis and readily misdiagnose it. Therefore, it was essential that it was diagnosed correctly. First, a detailed understanding of the patient’s case history was required. Patients with popliteus tendinitis usually had posterolateral pain in the knee joint for running downhill and participating in strenuous activities. Upon physical examination, the main finding was tenderness along the posterolateral joint line. Palpating while the patient sit up with the leg crossed in a “4” position was recommended. For the two specific physical examinations, the patient was asked to resist the examiner’s external rotation force on the tibia. These actions resulted in pain if the popliteus tendon was inflamed. Although more common medially, a popliteus cyst, lateral meniscus tear or lateral compartment articular damage presented a similar pattern of symptoms. If the physician was not sure about the diagnosis, the imaging examination (radiographs and MRI) might be helpful (16). In our study, there were 11 and 12 patients with increased intratendinous or myotendinous signals and fluid signals around the popliteus tendon in MR images, respectively. MRI had a vital role in the diagnosis and treatment management of popliteus tendinitis due to inherent difficulties in visualizing this region with arthroscopy and the challenge in detecting these injuries clinically.

Generally, the patients with popliteus tendinitis chose conservative therapy for their management. These patients required a reduction in sports activities, getting more rest, avoiding trauma or injury, and even wearing knee braces with 30° of knee flexion for 3 weeks (17). When popliteus tendinitis was accompanied by severe symptoms or injuries in other posterolateral side structures, there were different managements depending on the specific circumstance. In Eric’s study, a patient with a popliteus tendon injury received a steroid injection and conservative management with physical therapy (8). Petsche et al. suggested that most patients respond well to physical therapy and NSAIDs (5). Recalcitrant cases could require a local corticosteroid injection. In Blake’s study of patients with popliteus tendon tenosynovitis, the sheath was injected with 40 mg of methylprednisolone under arthroscopic guidance after joint irrigation (3). However, repeated corticosteroid injections resulted in a series of unwanted events, including a loss of tensile strength of the tendon, septic arthritis and a postinjection flare (7, 8, 18).

For most popliteus tendinitis patients, we recommend non-surgical therapy for those who had minor injuries. However, for patients whose symptoms were severe and not responsive to conservative therapy, the patients could undergo arthroscopic intervention. In our study, we chose an extreme lateral approach to obtain a better view of the relations of the popliteus tendon, tibial plateau and their trajectories. In addition, we also cleaned the loose body that was beside the popliteus hiatus with this approach (see the details on our previous studies) (19). In our cohort of patients, there were significant improvements in knee joint scores (Lysholm, Tegner, IKDC and VAS scores) after surgery compared with the preoperative data. Hence, arthroscopic treatment was the positive and effective clinical management in severe popliteus tendinitis patients.

In this article, we introduced a new extreme lateral approach to observe and manage popliteus tendonitis, which was accompanied by deformation of the popliteus hiatus. From our intraoperative findings, we observed that long-term chronic abrasion of the knee joint caused tissue degeneration injuries and inflammation of the popliteus tendon articular cavity. The patients felt discomfort and pain with this degeneration. Through arthroscopic observation and ablation, local inflammation and wear were effectively controlled. The postoperative symptoms were significantly relieved. After follow-up, the patients’ life quality was improved, and the knee function score increased significantly. From 2016 to 2020, only 15 patients required arthroscopic intervention. It was also proved that the incidence of popliteus tendinitis was low and conservative treatment played an important role in the treatment for it. Arthroscopic surgery was an effective method in recurrent patients or even patients with a more serious condition.

The present study has several limitations, however. Firstly, the study included the use of a small sample size. This was due to our institution’s strict inclusion criteria and lower incidence of popliteus tendinitis. Secondly, patient selection bias could not be avoided because many factors were self-reported, such as occupation, drugs. Thirdly, this study was a single-center study. Multi-center and large-sample studies are necessary in the future.

## Conclusion

Popliteus tendinitis is an unusual condition that causes pain in the posterolateral compartment of the knee joint. In our study, all the patients with popliteus tendinitis who were treated with arthroscopic popliteus tendon ablation via the auxiliary extreme lateral approach achieved satisfactory outcomes in terms of pain relief and improved function. We recommend that arthroscopic treatment be considered an option in cases of persistent and intractable popliteus tendinitis.

## Data Availability

The original contributions presented in the study are included in the article/Supplementary Material, further inquiries can be directed to the corresponding author/s.

## Supplementary Material

Supplementary Video S1 | Comprison of popliteus tendon before and after arthroscopic treatment

Supplementary Video S2 | Intra-articular trajectory of popliteus tendon under arthroscopy
